# Primary Gastric Alveolar Rhabdomyosarcoma: A Potent Mimicker of Neuroendocrine Carcinoma

**DOI:** 10.7759/cureus.69907

**Published:** 2024-09-22

**Authors:** Noriko Okuno, Kohji Takagi, Takahiko Nakajima, Haruka Fujinami, Kenichi Hirabayashi, Masakiyo Sasahara

**Affiliations:** 1 Department of Pathology, Faculty of Medicine, Academic Assembly, University of Toyama, Toyama, JPN; 2 Department of Diagnostic Pathology, Toyama University Hospital, Toyama, JPN; 3 Department of Diagnostic Pathology, Tonami General Hospital, Tonami, JPN; 4 Department of Endoscopy, Toyama University Hospital, Toyama, JPN

**Keywords:** alveolar rhabdomyosarcoma, gastric tumor, gastrointestinal pathology, rhabdomyosarcoma (rms), upper endoscopy

## Abstract

Alveolar rhabdomyosarcoma (ARMS) is a malignant tumor with skeletal muscle differentiation that usually occurs in soft tissues of the extremities or trunk. To date, only a few cases of primary gastric ARMS have been reported. Herein, we describe a case of ARMS in a man in his 80s and present a literature review. A submucosal tumor-like lesion was detected endoscopically. Histologically, the tumor cells exhibited a poorly differentiated morphology with hyperchromatic nuclei. Neuroendocrine markers, such as synaptophysin and CD56, were positive, and neuroendocrine carcinoma (NEC) was considered a differential diagnosis. However, both myogenic markers and OLIG2, which reflect FAX3 or FAX7 fusion, were positive, leading to a diagnosis of ARMS. Primary gastric ARMS is an extremely rare condition. Better awareness of this entity and its similarity to NEC is necessary for appropriate diagnosis and treatment.

## Introduction

Rhabdomyosarcoma (RMS) is a malignant soft-tissue tumor characterized by skeletal muscle differentiation [1]. However, these tumors rarely occur in the stomach. Among the four subtypes of RMS in the World Health Organization classification, primary gastric alveolar RMS (ARMS) is extremely rare [2, 3]. Herein, we report a case of primary gastric ARMS. The tumor consisted of round cells mimicking a neuroendocrine neoplasm (NEN), with positive staining for synaptophysin and CD56. Early detection led to a favorable prognosis. In addition, we briefly review the relevant literature.

## Case presentation

**Figure 1:**
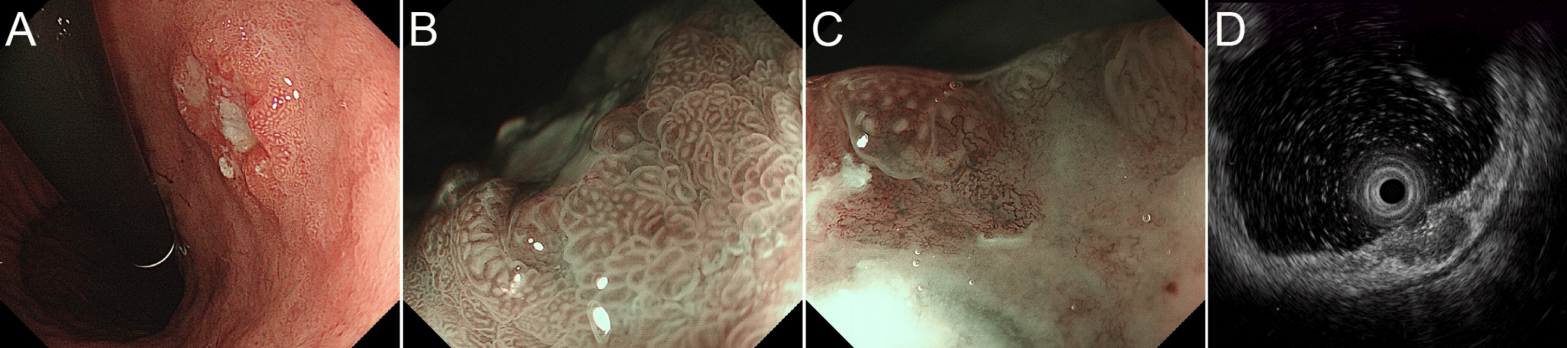
Endoscopic findings of the tumor. Endoscopically SMT-like lesion with ulcer was pointed out (A). NBI shows a lack of a microsurface (B) pattern and irregular microvessels (C). With EUS, the tumor shows a predominantly submucosal location (D).

The patient, a man in his 80s, underwent endoscopic submucosal dissection for a well-differentiated tubular adenocarcinoma one year previously. The tumor was located in the anterior wall of the stomach without any sarcomatous components. It was limited to the mucosa without vascular invasion, and the resection margin was negative. Endoscopic examination revealed a submucosal tumor (SMT)-like lesion with an ulcer located in the posterior wall distant from the previously treated lesion (Figure 1A). Magnifying narrow-band imaging (NBI) revealed a lack of microsurface patterns and irregular microvessels (Figure 1B-1C). Endoscopic ultrasonography (EUS) indicated that the tumor was primarily located in the submucosa (Figure 1D).

**Figure 2:**
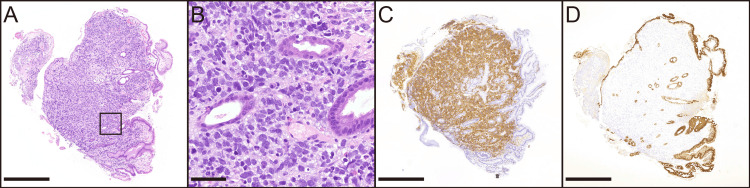
Histological findings of the biopsy specimen. Low-power image of hematoxylin and eosin staining reveals tumor growth in the lamina propria (A). The black box indicates the area enlarged in B. The high-power image of the biopsy specimen shows poorly differentiated tumor cells (B). Synaptophysin staining is positive for tumor cells in the interstitial space (C). CKAE1/AE3 staining is only positive for non-tumoral glandular epithelium (D). Scale bars indicate 500 μm in (A, C, D) and 50 μm in (B).

In the endoscopic biopsy specimen, round cells were distributed in a sheet-like pattern between the existing glands (Figure 2A). The tumor cells had enlarged nuclei with hyperchromatic chromatin and scant cytoplasm, suggesting poor differentiation (Figure 2B). Mitotic figures were also observed. These tumor cells were diffusely positive for the neuroendocrine markers synaptophysin and CD56 (Figure 2C). The Ki-67 labeling index was also high (around 90%). The patient was negative for chromogranin A expression. Therefore, gastric neuroendocrine carcinoma (NEC), which is a high-grade NEN, was suspected. However, negative staining for the epithelial markers CKAE1/AE3 and CAM5.2 made a definitive diagnosis difficult (Figure 2D).

**Figure 3:**
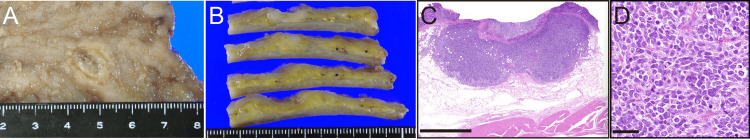
Macroscopic findings of the tumor. Gross appearance of the resected tumor (A). The cut surface shows the tumor located from lamina propria to submucosa (B). Hematoxylin and eosin staining. The ulcer lesion is correlated with necrosis (C). The tumor cells were poorly differentiated and showed sheet-like proliferation without fibrous septa (D). Scale bar indicates 2.5 mm in (C) and 50 μm in (D).

**Figure 4:**
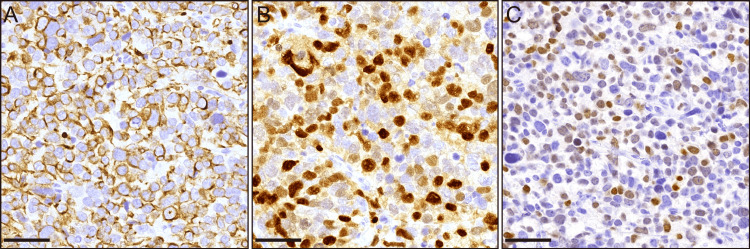
Immunohistochemical finginds of the resected tumor. High-power image of resected specimen. Immunohistochemically the tumor cells are positive for desmin (A), myogenin (B), and OLIG2 (C). Scale bars indicate 50 μm in (A-C).

A distal gastrectomy was performed. Consistent with endoscopic findings, an SMT-like lesion with an ulcer was observed (Figure 3A). The cross-section showed a solid white lesion extending from the mucosa to the submucosa (Figure 3B). Histologically, the central ulceration was associated with necrosis (Figure 3C). The viable areas consisted of poorly differentiated tumor cells, as observed in the biopsy specimen. The cells exhibited poor interconnectivity (Figure 3D). In addition to NEC, differential diagnoses included round cell sarcoma, such as RMS, lymphoma, and Ewing sarcoma. Fibrous septa were not observed. These tumor cells were also positive for synaptophysin and CD56 but negative for other neuroendocrine markers, including chromogranin A, INSM1, and SSTR2A. Additionally, the tumor was positive for desmin, myogenin, and MYOD1, suggesting skeletal muscle differentiation (Figure 4A-4B). Primary gastric RMS is extremely rare [2, 3], and the tumor was suspected to be a component of carcinosarcoma or a metastasis from other organs. However, despite sampling of the entire specimen, no carcinomatous components were identified. The only other tumor identified on imaging was in the prostate gland. It was histologically diagnosed as acinar adenocarcinoma based on the needle biopsy specimen. Overall, we concluded that the tumor was primary gastric RMS. OLIG2 is a specific marker of ARMS with PAX3/7-FOXO1fusion [4]. In the present case, OLIG2 was positive, leading to the diagnosis of ARMS (Figure 4C).

The tumor was completely resected, with no residual tumor. Lymph node metastasis was not observed. Given the patient’s underlying conditions, chronic heart failure, and renal failure, close follow-up without adjuvant therapy was planned. Four years after the surgery, there was no recurrence or metastasis.

## Discussion

RMS exhibits histological and immunohistochemical evidence of skeletal muscle differentiation [1]. RMS is classified into four histological subtypes: ARMS, embryonal RMS, spindle cell/sclerosing RMS, and pleomorphic RMS. The alveolar type constitutes approximately 40% of cases and involves not only the soft tissue, but also the perineal, paraspinal, and paranasal sinuses. It is typically characterized by rapid growth in the extremities; however, atypical locations may present with a range of symptoms, depending on the surrounding tissue involvement. Each RMS subtype displays distinct histological features. ARMS is characterized by round monomorphous cells with hyperchromatic nuclei [5]. Tumor cells are often attached to fibrous septa, although a solid subtype lacking these fibrous septa can be easily misdiagnosed. The tumor cells were variably positive for desmin, myogenin, and MYOD1. Genetically, ARMS exhibits PAX3-FOXO1 or PAX7-FOXO1 fusion [1].

Primary gastric RMS is a rare condition. ARMS is particularly rare, with only two cases reported to the best of our knowledge [2, 3]. In previous cases, similar central necrosis or ulcer formation has been reported for SMT-like lesions. Therefore, the endoscopic appearance observed in this case was considered characteristic of ARMS. Detailed descriptions of gastric ARMS incorporating NBI have not yet been reported. This is the first case to reveal the absence of a microsurface pattern and irregular microvessels in a gastric ARMS. These endoscopic features closely overlap with gastric NEC [6]. Furthermore, the sheet-like proliferation of poorly differentiated round cells, without fibrous septa as seen in this case, closely resembles the histology of NEC [7]. Immunohistochemical staining is critical for differentiating between the two, with positive myogenic and negative neuroendocrine markers. However, synaptophysin, a major neuroendocrine marker, has been reported to be positive in some ARMS cases [8]. One hypothesis is the association of PAX3 or PAX7 fusion with neurodevelopmental gene expression in ARMS [4].

RMS and NEN commonly present with nonspecific gastrointestinal symptoms. NEN, including NEC, occurs most frequently in the stomach among all organs, and the incidence of gastric NENs has increased 15-fold with advances in endoscopic techniques [7]. In contrast, ARMS in the stomach is extremely rare. Due to the similarities in endoscopic and histological features, it is important to avoid misdiagnosis of NEC. Although synaptophysin is an excellent neuroendocrine marker, it has been reported to be positive in other conditions such as malignant melanoma, angiosarcoma, and classical Hodgkin lymphoma [9]. Similarly, CD56, which was also positive in this case, is a marker for NEN but is less specific and can be positive in a variety of other tumors [10]. Therefore, when encountering a synaptophysin-positive poorly differentiated round cell tumor in the stomach, it is important to evaluate multiple and more specific neuroendocrine markers, such as chromogranin A, INSM1, and SSTR2A [10-12], as well as to rule out differentiation into other tissues, including skeletal muscle.

Surgery is the primary treatment for RMS [13]. ARMS is one of the few sarcomas that can metastasize to the regional lymph nodes [5]. Although all previous reports involved lymph node metastasis, this case was detected early and no lymph node metastasis was observed [2, 3]. Additionally, early detection allows R0 resection in this case, which is an independent favorable prognostic factor for RMS [13].

## Conclusions

In our case report on primary gastric ARMS, the patient had a favorable prognosis without adjuvant chemotherapy. Understanding the clinical and histologic features of ARMS is crucial for appropriate management and prognosis. There is a paucity of knowledge regarding early-detected primary gastric ARMS. Further accumulation of cases and studies on the necessity of postoperative adjuvant therapy and other treatments are required.
